# Chemotaxis, growth, and inter-species interactions shape early bacterial community assembly

**DOI:** 10.1093/ismejo/wraf101

**Published:** 2025-05-28

**Authors:** Estelle E Clerc, Jeremy E Schreier, Jonasz Słomka, Christa B Smith, He Fu, Justin R Seymour, Jean-Baptiste Raina, Mary Ann Moran, Roman Stocker

**Affiliations:** Department of Civil, Environmental, and Geomatic Engineering, Institute for Environmental Engineering, ETH Zurich, 8093 Zurich, Switzerland; Division of Geological and Planetary Sciences, California Institute of Technology, Pasadena, CA 91125, United States; Department of Civil, Environmental, and Geomatic Engineering, Institute for Environmental Engineering, ETH Zurich, 8093 Zurich, Switzerland; Department of Marine Sciences, University of Georgia, Athens, GA 30602, United States; Department of Biology, North Carolina Agricultural and Technical State University, Greensboro, NC 27411, United States; Climate Change Cluster, University of Technology Sydney, 15 Broadway, Ultimo NSW 2007, Australia; Climate Change Cluster, University of Technology Sydney, 15 Broadway, Ultimo NSW 2007, Australia; Department of Marine Sciences, University of Georgia, Athens, GA 30602, United States; Department of Civil, Environmental, and Geomatic Engineering, Institute for Environmental Engineering, ETH Zurich, 8093 Zurich, Switzerland

**Keywords:** microfluidics, growth, chemotaxis, microbial communities, community assembly

## Abstract

From the perspective of a marine copiotrophic bacterium, the surface ocean is a mosaic of exploitable hotspots of organic matter released from living and senescing phytoplankton. Bacterial success in exploiting this patchy microscale environment relies on sensing and swimming towards the hotspots, and upon arrival, growing on available substrates. However, the combined effect of chemotaxis and growth rate on bacterial community assembly has never been quantified. Here, we characterized chemotaxis and growth rate responses of seven representative copiotrophic marine bacteria to phytoplankton exometabolites, both for single species and for pairs of species. We compared these results to prediction of a mathematical null model of hotspot community assembly. Our results revealed that the bacterial strains exhibit diverse responses to phytoplankton metabolites, which can act as either signals, substrates, or both. Interactions between bacterial pairs resulted in chemotactic responses or growth rates different from model predictions in all of the 12 pairs tested (83% differed in chemotaxis, 33% in growth). These community dynamics indicate that inter-species interaction is another factor shaping early colonization of metabolite hotspots, with beneficial, detrimental, and neutral associations observed between bacterial species. Such complex ecological interactions impact chemotactic behaviors and growth rates of marine bacteria on resource hotspots, affecting their community compositions and associated functions relevant to the cycling of key elements including carbon.

## Introduction

Understanding the processes underpinning the assembly of microbial communities is one of the fundamental goals of microbial ecology. In the ocean, newly available substrates can arise from marine particles [1–3], excretion [4, 5], lysis events [6–8], or the slow exudation of photosynthates from phytoplankton cells [9–11]. These nutrient hotspots provide substrates to fuel the growth of many microorganisms whose catabolism influence the carbon balance between the ocean and the atmosphere [12–16]. The colonization of these hotspots and the subsequent development of microbial communities are influenced by two important factors: who gets there first, and who grows the fastest.

Chemotaxis—the ability of motile microorganisms to direct their movements in response to chemical gradients—is an important behavior allowing cells to home in on nutrient hotspots and may determine the identity of the early colonizers [17, 18]. Yet the success of a foraging bacterium relies not only on finding a source of organic matter, but also on its ability to grow rapidly on the substrates available [18]. Compounds released in nutrient hotspots may act as signals that attract microorganisms [16, 19, 20], as substrates for microbial growth [13, 14, 21–24], or both [25–30]. Experiments investigating bacterial community assembly on single carbon sources [26, 31] have identified modular assembly of many coexisting taxa, often triggered by cross-feeding of metabolic secretions [32]. It is therefore likely that interactions between colonizers (either beneficial or detrimental) may also affect the outcome of chemotaxis and growth. Although the effect of chemotaxis, growth, and inter-species interactions have each been studied on their own, their combined effects on community assembly have not been characterized.

Here, we address how bacterial chemotaxis toward phytoplankton metabolites and their subsequent catabolism influences assembly in simple two-species model communities. To this end, we first measured the individual chemotactic strengths [33] and growth rates of seven bacterial strains (previously isolated from coastal seawater enriched with phytoplankton exometabolites [31]) in response to dimethylsulfoniopropionate (DMSP) and spermidine, two important phytoplankton exometabolites [34, 35]. We then mimicked the early phase of bacterial community establishment in metabolite hotspots by conducting experiments with pairs of bacterial species and monitoring subsequent community composition following chemotaxis and growth on these metabolites. Using a mathematical null model parameterized with the chemotaxis and growth results for each species in isolation, we predicted the composition of the two-species communities in the absence of inter-species interactions. Comparing results of this null model with experimental outcomes allowed us to identify the role of inter-species interactions in chemotaxis and growth, and ultimately in the early assembly of bacterial communities.

## Materials and methods

### Bacterial strains

Alphaproteobacteria (*Phaeobacter* sp. HF9A, *Celeribacter* sp. HF31, *Thalassospira* sp. HF15) and Gammaproteobacteria (*Vibrio diazotrophicus* HF9B, *Vibrio* sp. HF57, *Pseudoalteromonas* sp. HF66, *Vibrio hepatarius* HF70) were isolated previously from bacterial enrichment communities used to study the effect of known marine phytoplankton exometabolites on bacterial community assembly [31]. These strains have fully sequenced genomes [36] and are motile in rich medium. Bacteria were grown from cryostocks on ½ YTSS (2.0 g/L yeast extract and 1.25 g/L tryptone) agar plates (1.5%) prepared with artificial seawater (20 g/L Instant Ocean, Spectrum Brand) and incubated at 27°C overnight. A single colony per strain was inoculated in strain-specific media (see below) optimized to promote bacterial motility. Cultivation protocols were established for each strain to optimize bacterial motility. All strains except *V. hepatarius* HF70 and *Thalassospira* sp. HF15 were inoculated into 1/20 YTSS. *V. hepatarius* HF70 was inoculated into ½ YTSS and *Thalassospira* sp. HF15 was inoculated in 1/10 Difco 2216 Marine Broth (BD Diagnostics). Liquid cultures were grown at 27°C for 20 h in a shaking incubator. Bacterial motility was confirmed by microscopy and a subsample (100 μl) was stained with SYBR Green I (ThermoFisher) to determine cell concentrations using a CytoFLEX S flow cytometer (Beckman Coulter) recording side scatter and FITC fluorescence.

### Chemotaxis assays

Chemotaxis was characterized using the In Situ Chemotaxis Assay (ISCA; [33, 37]), a microfluidic device consisting four rows of five micro-wells (110 μl each) that were individually loaded with one of ten 0.2 μm filter sterilized chemoattractants (1 mM in artificial seawater): DMSP (synthesized following the protocol of Chambers et al., 1987 [38]), spermidine (Sigma-Aldrich), ectoine (Sigma-Aldrich), 2,3-dihydroxypropane-1-sulfonate (DHPS, synthesized by K. Denger and A. Cook (Konstanz, Germany)), trimethylamine (TMA, Sigma-Aldrich), glycolate (Sigma-Aldrich), ribose (Sigma-Aldrich), isethionate (Sigma-Aldrich), xylose (Sigma-Aldrich) and glutamate (Sigma-Aldrich). In an ISCA, each row of five wells served as technical replicates for an individual chemoattractant. Each chemoattractant was deployed simultaneously in three ISCA replicates. Chemicals were inoculated in ISCA wells using a sterile 1 ml syringe (Codan, USA) and needle (27G, Henke Sass Wolf). Filtered artificial seawater was used as a negative control in each ISCA to account for cells entering wells by random motility.

Prior to chemotaxis experiments, bacteria were grown and enumerated as above. ISCAs were incubated in artificial seawater inoculated with 10^6^ bacteria ml^−1^ for 1 h, a density and timescale ecologically relevant as it mimics coastal surface seawater [39] and specific nutrient sources in the ocean [40]. After incubation, contents of the ISCA wells were recovered using a sterile syringe and needle, and technical replicates were pooled [41]. Chemotactic indices were determined by measuring cell concentrations in experimental samples relative to the filtered artificial seawater control.

### Chemotaxis assay to HF66 supernatant

To further understand the increased chemotactic response of *Thalassospira* sp. HF15 in presence of *Pseudoalteromonas* sp*.* HF66, a specific ISCA experiment was designed. An overnight culture of *Pseudoalteromonas* sp. HF66 was washed twice in sterile artificial seawater (Sigma Sea Salts), resuspended at 10^6^ cells ml^−1^ in seawater containing 1 mM DMSP, incubated at room temperature for 1 h, and then sequentially filtered through 0.2 μm Sterivex filter (Millipore) and 0.02 μm Anotop filter (Whatman). The resulting spent medium was used as a chemoattractant alongside DMSP (1 mM unincubated) in the ISCA, and the bulk seawater was inoculated with 10^6^ cells ml^−1^ of *Thalassospira* sp. HF15. After 1 h, samples were retrieved, and cells were enumerated by flow cytometry.

### Substrate growth tests

To assess the ability of the bacterial strain to grow on the selected metabolites, the strains were individually inoculated into ½ YTSS medium and grown overnight at 30°C in a shaking incubator. Cells were pelleted at 4000 × g, washed three times, and resuspended to an OD_600_ of ~0.01 in Marine Basal Medium [42] supplemented with 3 mM ammonium and each phytoplankton metabolite at 12 mM carbon. Cultures were grown in a 96-well plate in triplicate in a Synergy H1 microplate reader (BioTek) at 30°C with constant shaking and OD_600_ was measured every hour. Maximum growth rates were determined as the slope of the linear portion of the natural log-transformed data [43].

### Chemotaxis-growth competition assay

Bacterial strains were prepared as above for chemotaxis assays. ISCA triplicates containing DMSP and spermidine were prepared as above and were incubated for 1 h in artificial seawater with *Pseudoalteromonas* sp. HF66 and one of six other bacterial strains in an equal mixture of 10^6^ total bacteria cells ml^−1^. While we aimed to inoculate bacterial pairs at equal concentrations, the clumping phenotype of *Pseudoalteromonas* sp. HF66 in rich medium led to an overrepresentation of this strain at the start of the experiment. To account for this, we quantified initial strain abundances using 16S rRNA gene amplicon sequences and used these values for normalization in our chemotaxis modeling predictions (Supplementary Notes). Since clumps were ~160 times smaller than the ISCA well ports (Fig. S1), they could not obstruct entry to the device or hinder individual bacteria from migrating towards the nutrient source.

At the start of the experiment, a 200 μl sample of the bulk artificial seawater was flash frozen in liquid nitrogen to determine the relative abundance of each strain prior to chemotaxis using 16S rRNA gene amplicon sequencing. Following the chemotaxis assay, samples were retrieved from the ISCA wells, technical replicates were pooled, and each sample was split into three subsamples for 16S rRNA gene amplicon sequencing (200 μl), flow cytometry (50 μl), and for the growth assay (250 μl). Subsamples for the growth assay were transferred to 15 ml tubes and incubated at 27°C in a shaking incubator. After 24 h of growth, samples were collected for flow cytometry and 16S rRNA gene amplicon analysis.

### DNA extraction, sequencing, and 16S rRNA gene amplicon analysis

Samples were collected from paired chemotaxis-growth assays from the bulk artificial seawater prior to chemotaxis, from ISCA wells after 1 h of chemotaxis, and from cultures after the 24 h grow-out. In short, DNA was extracted using a physical lysis protocol optimized for low DNA input [44]. Samples were mixed with lysis buffer (potassium hydroxide + dithiothreitol) and incubated at room temperature for 10 min, at −80°C for 10 min, then at 55°C for 5 min, followed by the immediate addition of stop buffer (Tris–HCl). Extracted DNA was cleaned with AMPure XP beads (Beckman Coulter), eluted in nuclease-free water and stored at −20°C. The 16S rRNA gene V4-V5 hypervariable region was amplified using primers 515F-Y/926R [45], and libraries were constructed using the KAPA Hyper Prep Kit (Kapa Biosystems, Wilmington, MA). 16S rRNA gene amplicons were sequenced at the Georgia Genomics and Bioinformatics core using a MiSeq System (Illumina) single-end 150 base pair flow cell. Forward reads of amplicon sequences were processed in R with dada2 [46] using a standard pipeline (https://benjjneb.github.io/dada2/tutorial.html) with taxonomy assigned using SILVA 138.2.

To transform the 16S rRNA gene amplicon counts to cell abundances, we established three mock microbial communities by mixing different concentrations of each bacterium with known optical density (OD_600_ = 0.07) and cell counts (~1.2 × 10^5^ cells). DNA was extracted, 16S rRNA gene amplicon libraries were constructed and sequenced as previously reported. Given the total library sizes are not equal, we normalized the total reads of each library to a million. In all seven strains, we found that the normalized counts of amplicon sequence variant (ASV) are linear (*R*^2^ > 0.97) to the cell counts. These data validated the robustness of 16S rRNA gene amplicon sequencing, allowing us to calculate cell abundances based on their 16S rRNA gene amplicon counts.

### Data analysis

Statistical analyses and data display were performed in R 4.3.0 using ggplot2, vegan, ggpubr, dplyr, tidyr, data.table, and tibble, and maximum growth rates were calculated using Excel 2019.

## Results and discussion

### Phytoplankton exometabolites can act as signals and/or substrates for marine bacteria

**Figure 1:**
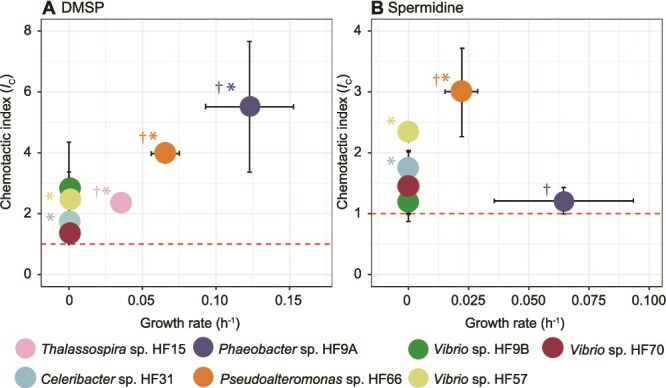
Chemotactic index (*I_C_*) and maximum growth rate of seven bacterial strains on two phytoplankton metabolites, DMSP (A) and spermidine (B). An *I*_C_ significantly greater than 1 (dashed line) represents chemotactic attraction and is indicated by a color-coded asterisk (Datasets S2-S3, t-tests, *P* ≤ 0.05). A dagger highlights significant growth within 24 h (Dataset S1, t-tests, *P* ≤ 0.05). The standard deviation (*n* = 3) is displayed for all means, and in some cases falls within the symbols.

To investigate the impact of individual phytoplankton metabolites on bacterial community assembly, we first measured the chemotaxis and growth rates of seven bacterial strains in response to the organic sulfur compound DMSP and the polyamine spermidine. These two compounds are widely produced by phytoplankton, constitute some of the most abundant sources of reduced carbon [34, 35], sulfur [47, 48], and nitrogen [49], and are known chemoattractants for marine bacteria [29, 50, 51]. The seven bacterial strains tested were previously isolated from coastal seawater enriched with phytoplankton exometabolites [31] and included three marine Alphaproteobacteria (*Thalassospira* sp. HF15, *Celeribacter* sp*.* HF31, *Phaeobacter* sp. HF9A) and four marine Gammaproteobacteria (*Pseudoalteromonas* sp*.* HF66, *V. diazotrophicus* HF9B, *Vibrio* sp. HF57, *V. hepatarius* HF70). The chemotactic response of each strain was quantified using the In Situ Chemotaxis Assay (ISCA), a microfluidic device consisting of a set of micro-wells, each filled with a metabolite and connected to the external environment by a port allowing for both the diffusion of metabolites into the surrounding seawater, and the migration of chemotactic bacteria into the well [33]. We used the chemotactic index (*I*_C_)—the cell number in the ISCA wells containing the metabolite divided by the cell number in control wells containing filtered artificial seawater, both measured after one hour—to quantify the strength of the chemotactic responses (Fig. 1). Growth rates of bacterial strains were independently calculated based on a 24 h incubation of each strain on each metabolite [43].

DMSP played a role as both a signal (i.e. chemoattractant) and a substrate for three strains—*Thalassospira* sp. HF15, *Phaeobacter* sp. HF9A, and *Pseudoalteromonas* sp*.* HF66 (Fig. 1A, t-tests, *P* ≤ 0.05, Dataset S1-S2). DMSP functioned only as a signal for two strains (Fig. 1A, Dataset S1-S2, t-tests, *P* ≤ 0.05). In comparison, spermidine displayed a dual role (signal and substrate) only for *Pseudoalteromonas* sp*.* HF66 (Fig. 1B, Datasets S1, S3, t-tests, *P* ≤ 0.05). In two strains, spermidine acted solely as a signal and in one strain solely as a substrate (Fig. 1B, Datasets S1, S3, t-tests, *P* ≤ 0.05).

### Chemotactic bacterial strains influence each other’s chemotaxis

After characterizing the chemotaxis and growth rate of each strain in response to DMSP and spermidine, we tested the responses of pairs of strains, introducing the opportunity for interactions. The experiment measured the chemotactic arrival of the two strains onto a simulated nutrient hotspot and their subsequent growth on the hotspot. Each of the other six strains was paired with *Pseudoalteromonas* sp*.* HF66, the only strain that exhibited chemotaxis and growth on both DMSP and spermidine (Fig. 1, Datasets S1-S3, t-tests, *P* ≤ 0.05). Bacterial pairs inoculated at densities comparable to coastal surface seawater [39] were allowed to chemotax to ISCA wells for 1 h, after which the well contents were incubated for 24 h (Fig. S2).

**Figure 2:**
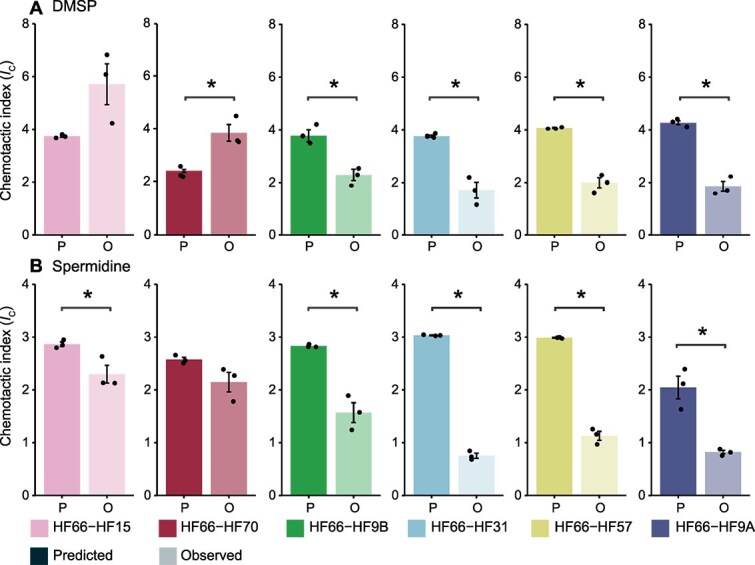
Chemotactic index (*I*_C_) of paired strains on (A) DMSP and (B) spermidine after a 1 h ISCA assay. The predicted *I*_C_ calculated from a mathematical model (marked as a “P”; Supplementary Note 1) was compared to the observed *I*_C_ determined by 16S rRNA gene amplicon sequencing (marked as a “O”). Significant differences between predicted and observed chemotactic indices are indicated by an asterisk (Fig. 2, Dataset S4, ANOVA, *P* ≤0.05). Colored bars display average *I*_C,_ bars denote the standard deviation, and points are individual replicates (*n* = 3).

We analyzed the chemotactic index of each pair to determine if inter-species interactions can influence the chemotactic response. The observed *I*_C_ value of a species pair (i.e. the total chemotactic response regardless of species identity) was compared to the prediction from a mathematical null model of chemotaxis, which assumes that each strain performs chemotaxis independently of the other strain (Fig. 2, Dataset S4, Supplementary Note 1). Significant differences in predicted versus observed chemotactic performances occurred in 10 out of 12 experiments (i.e. six pairs tested for two molecules; Fig. 2, Dataset S4, ANOVA, *P* ≤0.05). The chemotactic response of *Pseudoalteromonas* sp*.* HF66 with *V. hepatarius* HF70 in response to DMSP was significantly stronger than predicted by the null model, whereas for the other nine pairs they were significantly weaker (Fig. 2, Dataset S4, ANOVA, *P* ≤ 0.05), suggesting antagonistic or inhibitory interactions between chemotaxers.

To better understand the role of each strain in the combined chemotaxis response of the pair, we used 16S rRNA gene amplicon data after the chemotaxis assay to infer the *I*_C_ of each member of the pair (Figs. S3-S4, Dataset S5) and compared these data to the *I*_C_ to each member in isolation (Fig. 1). Of the 12 pairs tested, half displayed significantly altered chemotaxis for one strain in the pair compared to that strain alone, three displayed significantly altered chemotactic response for both strains in the pair, and three displayed no difference for either strain (Figs. S3-S4, Dataset S6, ANOVA, *P* ≤0.05). The direction of change in chemotactic responses were considered in light of ecological outcomes of microbial interactions [52, 53]: detrimental (−), neutral (0), or beneficial (+). Detrimental outcomes, defined here as a significantly lower chemotaxis response than when in isolation for at least one strain of the pair, were the most common (seven of 12 experiments). In four of these pairs, one strain was hampered and the other unchanged (−/0). For example, the *I*_C_ of *Pseudoalteromonas* sp*.* HF66 towards DMSP was two times lower when paired with *V. diazotrophicus* HF9B than when alone. In two other pairs, both strains exhibited significantly lower chemotaxis when paired relative to in isolation (−/−). This occurred for the *Pseudoalteromonas* sp. HF66/*Celeribacter* sp. HF31 and *Pseudoalteromonas* sp*.* HF66/*Vibrio* sp*.* HF57 pairs towards spermidine (Fig. S4, Dataset S6, ANOVA, *P* ≤ 0.05).

There were three instances in which one strain of the pair increased its chemotactic response relative to when alone, and all occurred in the DMSP assays. For example, *Pseudoalteromonas* sp. HF66 displayed a 2.5-fold chemotaxis response increase (Fig. S3F, Dataset S6, ANOVA, *P* ≤0.05) whereas *Phaeobacter* sp*.* HF9A exhibited a five-fold reduction (+/−). In two other instances, the chemotactic responses of both *Thalassospira* sp. HF15 and *V. hepatarius* HF70 significantly increased when paired with *Pseudoalteromonas* sp. HF66, whereas HF66 chemotaxis remained unaffected (0/+) (Fig. S3A-B, Dataset S6, ANOVA, *P* ≤ 0.05). As these outcomes suggested that inter-species interactions can stimulate chemotaxis, we tested whether spent medium from a *Pseudoalteromonas* sp*.* HF66 culture growing on DMSP for 1 h served as a chemoattractant for *Thalassospira* sp*.* HF15. The resulting *I*_C_ for *Thalassospira* sp. HF15 was 40% stronger towards the filtrate compared to DMSP (Fig. S5, Dataset S8; ANOVA, *P* ≤0.05), suggesting that metabolites released by HF66 enhanced the chemotactic response of *Thalassospira* sp. HF15. Overall, our results reveal that ecological interactions between bacterial species chemotaxing towards nutrient hotspots are common and occur rapidly enough to alter the behavior of other bacteria over timescales relevant to the quest for transient nutrient hotspots.

### Growth capability is a predictor of community assembly

**Figure 3:**
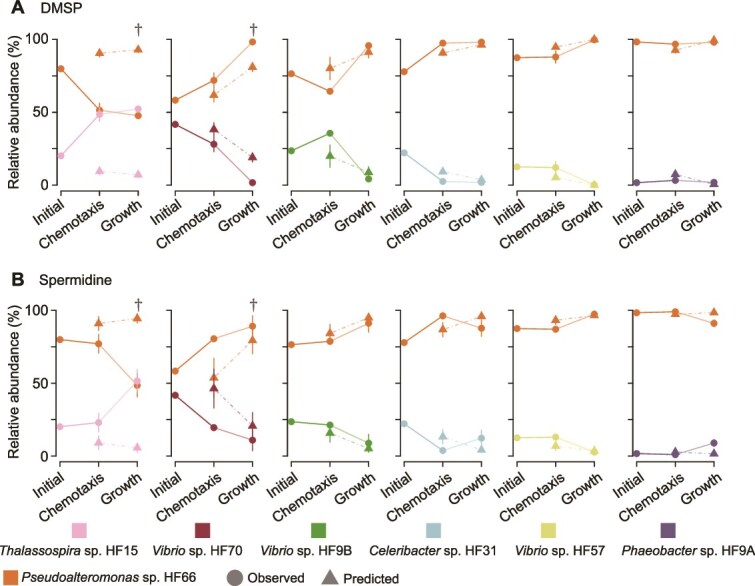
Relative abundance of *Pseudoalteromonas* sp*.* HF66 and a paired second strain in artificial seawater at the time of inoculation (initial), after 1 h of chemotaxis and after 24 h of growth on either (A) DMSP or (B) spermidine (Dataset S5). Deviations between observed (circles) and predicted (triangles) community composition greater than 10% at the end of the growth phase are indicated by dagger symbols (Dataset S9).

We next addressed how the strains’ growth on metabolite hotspots following chemotaxis influenced bacterial community assembly. The mathematical null model of chemotaxis was expanded to include the growth phase subsequent to chemotaxis, using growth rates of each strain measured on DMSP and spermidine (Fig. 1, Supplementary Note 2). Comparison of null model results with measured growth allowed us to compute the percent deviation between the predicted and observed community composition following the chemotaxis and growth phases (Dataset S9). Despite differences in the initial abundance of strains, deviations between experimental results and null model predictions greater than 10% provided a conservative approach to identifying inter-species interactions (Supplementary Note 2, Fig. S6). Cases where the predicted abundance deviated from the observed abundance by less than 10% were considered strong matches to the null model, indicating that strain interactions had no or only minor effects on final community composition. Conversely, cases where the predicted abundance deviated from the observed abundance by more than 10% were considered poor matches, indicating that strain interactions had a major effect on final community composition. We found that the expanded null model successfully predicted final species’ contributions to the community in 67% of tests (8 out of 12 pairs; four on DMSP and four on spermidine, Fig. 3, Dataset S9). Therefore, we concluded that pairwise bacterial interactions during the growth phase were sufficiently weak in the majority of pairs to allow the null model to accurately estimate community compositions.

In 33% of the tested pairs, substantial deviations between predicted and observed community composition indicated interactions between strains that were sufficient to alter the outcome of community assembly. The greatest deviations were observed in the *Pseudoalteromonas* sp*.* HF66 and *Thalassospira* sp. HF15 pair on both DMSP and spermidine (Fig. 3A; Dataset S9, deviation up to 45%), with HF15 abundance increasing from a quarter of the initial community to half of the final community, while the model predicted a decrease (Fig. 3A-B) (Dataset S9). The deviations had appeared already during the chemotaxis phase and were maintained or accentuated during the growth phase. All bacterial pairs tested exhibited inter-species interactions during either the chemotaxis phase, the growth phase, or both (Figs. S3-S4, Dataset S6, ANOVA, *P* ≤ 0.05), indicating that inter-species interactions are prevalent in the race between bacteria to colonize new nutrient hotspots.

### Marine exometabolites have strain-specific roles in the ocean microbiome

**Figure 4:**
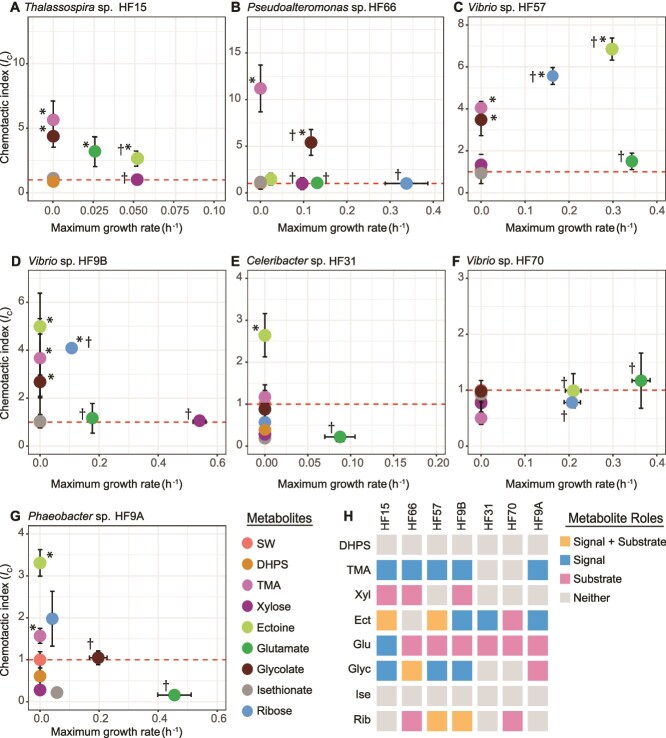
(A-G) Chemotactic index (*I*_C_) and maximum growth rate of seven bacterial strains on eight phytoplankton metabolites (plus an artificial seawater control, “SW”). An *I*_C_ value significantly greater than 1 (dashed line) represents attraction, and significantly lower than 1 represents repulsion, with asterisks denoting a response significantly greater than 1 (Dataset S10, t-tests, *P* ≤ 0.05). A dagger highlights significant growth within 24 h (Dataset S1, t-tests, *P* ≤ 0.05). The standard deviation (*n* = 3) is displayed for all points, and in some cases falls within the symbol. (H) Summary map of metabolite roles by strain. Among the 56 chemotaxis-growth assays with seven strains and eight additional metabolites, five metabolites were both signals and substrates (9%), 12 were signals (21.4%), 13 were substrates (23.2%), and 26 did not elicit any response (46.4%). SW: Artificial seawater, DHPS: 2,3-dihydroxypropane-1-sulfonate, TMA: trimethylamine, Xyl: xylose, Ect: ectoine, Glu: glutamate, Glyc: glycolate, Ise: isethionate, Rib: ribose.

DMSP and spermidine represent specific examples of the complex chemical seascape of the surface ocean [16, 54, 55]. We therefore extended the chemotaxis and growth assays to include eight additional representative marine phytoplankton metabolites and characterized their chemical roles (signal, substrate, both, or none) in community assembly. The eight exometabolites spanned multiple chemical classes, including amino acids, amino acid derivatives, organic acids, and organic sulfur compounds (Fig. 4A-G).

Ecological roles identified for the test metabolites differed among strains (Fig. 4A-G, Datasets S1, S10), in agreement with previous observations [19, 29]. Out of the 56 chemotaxis and growth assays, the tested metabolite acted as both a signal and a substrate in five cases (9%), only as a signal in 12 cases (21.4%), only as a substrate in 13 cases (23.2%), and as neither a signal nor substrate in 26 cases (46.4%) (Fig. 4H, Datasets S1, S10, t-tests, *P* ≤ 0.05). Ectoine, glycolate, glutamate, and ribose acted as substrates, signals or both depending on the strain (Fig. 4A-G, Datasets S1, S10). For four other metabolites, the ecological outcomes were consistent across the seven strains. For instance, trimethylamine (TMA) was not used as a substrate by any of the strains but attracted five strains (Fig. 4A,B,C,D,G, Datasets S1, S10, t-tests, *P* ≤ 0.05). Conversely, xylose functioned as a substrate for three strains (Fig. 4A,B,D, Dataset S1, t-tests, *P* ≤ 0.05) and did not attract any. The sulfonate metabolites 2,3-dihydroxypropane-1-sulfonate (DHPS) and isethionate neither elicited a chemotactic response nor supported growth in any of the strains (Fig. 4A-G, Datasets S1, S10, t-tests, *P* < 0.05). These results show that phytoplankton exometabolites can affect bacterial success in colonizing hotspots, but the outcome depends on the bacterium. These results further highlight that molecules used as signals are not necessarily used as substrates for growth by the bacteria. Instead, these signal molecules may indicate the presence of nutrient hotspots where a wide range of metabolites are available for bacterial uptake.

## Conclusions

Chemotaxis and growth are important phenotypes influencing bacterial success in all environments where nutrient availability is patchy [40, 56]. Previous work has typically decoupled these two factors by investigating the strength of chemotaxis towards specific cues [19, 37] or the growth rate on specific substrates [31, 57] in isolation. By considering the joint effect of chemotaxis and growth on community assembly, we systematically captured the coupling of these two phenotypes. A mathematical null model parameterized with observational data of chemotaxis and growth when only one bacterial species was present was used to predict community assembly outcomes for species pairs. This approach revealed that bacterial community assembly in nutrient hotspots is not always predictable from ecological behaviors when alone, suggesting a key role for interactions between strains. Interactions were found to occur for all bacterial pairs tested either during the chemotaxis phase (Fig. 2) or the growth phase (Fig. 3). Interactions were more frequently detrimental, although some were beneficial with evidence for mediation by released metabolites. While we studied these dynamics at a single temperature, future work should explore how varying temperatures, including those in colder ocean regions or at optimal bacterial growth conditions, may affect competitive outcomes—especially in the context of ocean warming.

Nutrient patches span a wide range of lifetimes, from minutes for those created by microbial cell lysis and copepod excretion [6], to hours or days for those associated with marine snow and phycospheres [1, 11]. Although our study measured bacterial growth over 24 hours, shorter-term exposure to nutrient-rich microenvironments still elicits growth and can further stimulate bacterial activity via transient metabolic upregulation [25, 27]. Additionally, DMSP and spermidine are enriched in longer-lived hotspots such as particles and phycospheres [34, 35]. Overall, the 24-hour incubation period considered here is relevant for assessing bacterial chemotaxis, growth, and inter-species interactions in ecologically important microenvironments.

A common goal of ecology is to understand the processes by which bacterial species disperse and interact to form local communities centered around resources. Priority effects stemming from the order and timing of a bacterial population’s arrival to ephemeral nutrient hotspots paves the way for the ensuing ecological succession [58]. The approach used here, merging chemotaxis and growth assays, falls broadly within microbial landscape ecology, which considers how the spatial and temporal availability as well as composition of nutrients affect species assemblages [59]. The chemotactic and growth responses of the bacteria tested in this study exemplify the roles metabolites can play as signals and/or substrates; roles that differ between bacterial strains and are subject to further modification from ecological interactions. The prevalence of detrimental outcomes observed here suggests that bacteria can inhibit the chemotactic response of potential competitors, reducing the rate at which strains may arrive at a nutrient hotspot. Beneficial outcomes, on the other hand, illustrate how quickly niche space can be modified in a manner that facilitates the arrival and growth of others. Together, resources that function as substrates and signals and ensuing inter-species interactions provides a framework for determining how the heterogeneous distribution of carbon and nutrients affects the rate of remineralization in the ocean’s patchy microenvironments.

## Supplementary Material

Clerc_and_Schreier_SupplementaryInformation_Final_wraf101

Clerc_and_Schreier_Datasets_Final_wraf101

## Data Availability

The amplicon sequencing data (16S rRNA genes) derived from the competition assays have been deposited on NCBI under the BioProject accession PRJNA1234665. Monte Carlo simulations creating cumulative distribution functions were done in MATLAB to measure the model predictions’ uncertainty using a custom code (available on https://doi.org/10.5281/zenodo.12210966). Source data are provided with this paper.
