# Supplementary material for: Chemotaxis, growth, and inter-species interactions shape early bacterial community assembly
Source: ISME J. 2025 May 28;19(1):wraf101. doi: 10.1093/ismejo/wraf101 (PMC12202767; doi:10.1093/ismejo/wraf101)
Supplement: Clerc_and_Schreier_SupplementaryInformation_Final_wraf101 [file clerc_and_schreier_supplementaryinformation_final_wraf101.docx]

**Supporting Information for**

**Chemotaxis, growth, and inter-species interactions shape early bacterial community assembly**

Estelle E. Clerc^1^*, Jeremy E. Schreier^2^*, Jonasz Słomka^1^, Christa B. Smith^3^, He Fu^4^, Justin R. Seymour^5^, Jean-Baptiste Raina^5^, Mary Ann Moran^3#^ & Roman Stocker^1#^

^1^ Institute for Environmental Engineering, Department of Civil, Environmental, and Geomatic Engineering, ETH Zurich, Zurich, Switzerland

^2^ Division of Geological and Planetary Sciences, California Institute of Technology, Pasadena, CA, USA

^3^ Department of Marine Sciences, University of Georgia, Athens, GA, USA

^4^ Department of Biology, North Carolina Agricultural and Technical State University, Greensboro, NC, USA

^5^ Climate Change Cluster, University of Technology Sydney, Broadway, New South Wales, Australia

^#^ Correspondence to: [mmoran@uga.edu](mailto:mmoran@uga.edu) and [romanstocker@ethz.ch](mailto:romanstocker@ethz.ch)

**This file includes:**

Supporting Text (Supplementary Notes)

Figure Legends S1 to S7

Legends for Datasets S1 to S10

**Other supporting materials for this manuscript include the following:**

Datasets S1 to S10

**Supporting Information Text**

**Supplementary Notes**

**Supplementary Note 1: Null model**

For model predictions, we assume that:

1. The ISCA is introduced into a bacterial culture at time $t=0$. The culture contains bacteria with concentration $c$, while the ISCA wells do not contain bacteria at $t=0$, only substrates that diffusive outside of the ISCA wells and attract (or repel) bacteria.
2. The ISCA is deployed in the culture for a time $T_{exp}$.
3. The number of bacteria in each chemoattractant and artificial seawater negative control has been determined.

Assuming that the timescale of the experiments $T_{exp}$ is much shorter than the time needed for the concentration of bacteria in the wells to equilibrate with the outside concentration (which is generally warranted), the number of bacteria in a given well after time $T_{exp}$is:

$N=\Gamma T_{exp}c$

where $\Gamma$ is the encounter kernel that quantifies the rate at which bacteria in the culture encounter the well. $\Gamma$ depends on bacterial motility, chemotaxis strength, well geometry and (in general, but not in our experiments) external flow.

**Chemotactic index**

We consider two wells: a control well filled with artificial seawater and a well containing chemoattractant $A$. Assuming that the two wells are on the same ISCA, then the experiment lasts for the same amount of time for both wells and the bacterial concentration in the surrounding culture is the same for both wells. The number of bacteria in each well is then:

$N_{control}=\Gamma_{control}T_{exp}c, and$

$N_{A}=\Gamma_{A}T_{exp}c.$

The chemotactic index $IC_{A}$ for chemical $A$ is then defined as:

$IC_{A}=\frac{N_{A}}{N_{control}}=\frac{\Gamma_{A}}{\Gamma_{control}}.$

**Null model of chemotaxis for a mixture of two strains** $\alpha$ **and** $\beta$

We consider the case in which we have two bacterial strains $\alpha$ and $\beta$ and we measured their chemotactic indices $IC_{A}^{\alpha}$ and $IC_{A}^{\beta}$ for chemical $A$. The ISCA is then placed into a bacterial culture containing a mixture of both strains.

Assuming the two strains do not interact, we can calculate the resulting chemotactic index $IC_{A}^{\alpha\beta}$ for the mixture with the following assumptions. Let $c^{\alpha\beta}$ be the concentration of the bacterial culture containing both strains. Assuming the culture contains fraction $f$ of bacteria from strain $\alpha$ and fraction $1-f$ of bacteria from strain $\beta$, the concentration of strain $\alpha$ is $c^{\alpha}=fc^{\alpha\beta}$ and the concentration of strain $\beta$ is $c^{\beta}=(1-f)c^{\alpha\beta}$. Since the two strains do not interact, we can derive equations for the numbers of cells of each strain that are in the control and the well containing chemical A, respectively:

$N_{control}^{\alpha} =\Gamma_{control}^{\alpha}T_{exp}c^{\alpha}, and N_{A}^{\alpha}=\Gamma_{A}^{\alpha}T_{exp}c^{\alpha} N_{control}^{\beta} =\Gamma_{control}^{\beta}T_{exp}c^{\beta}, and N_{A}^{\beta}=\Gamma_{A}^{\beta}T_{exp}c^{\beta} (1)$

In the control well, the total number of cells is thus $N_{control}^{\alpha}+N_{control}^{\beta}$ and in the well containing chemoattractant $A$ the total number of cells is $N_{A}^{\alpha}+N_{A}^{\beta}$. The expected chemotactic index is then:

$IC_{A}^{\alpha\beta}=\frac{N_{A}^{\alpha}+N_{A}^{\beta}}{N_{control}^{\alpha}+N_{control}^{\beta}}=\frac{\Gamma_{A}^{\alpha}T_{exp}c^{\alpha}+\Gamma_{A}^{\beta}T_{exp}c^{\beta}}{\Gamma_{control}^{\alpha}T_{exp}c^{\alpha}+\Gamma_{control}^{\beta}T_{exp}c^{\beta}}=\frac{\Gamma_{A}^{\alpha}c^{\alpha}+\Gamma_{A}^{\beta}c^{\beta}}{\Gamma_{control}^{\alpha}c^{\alpha}+\Gamma_{control}^{\beta}c^{\beta}} .$

Using the definitions for the chemotactic indices measured on each of the two strains separately, namely $\Gamma_{A}^{\alpha}=IC_{A}^{\alpha}\Gamma_{control}^{\alpha}$ and $\Gamma_{A}^{\beta}=IC_{A}^{\beta}\Gamma_{control}^{\beta}$, as well as $c^{\alpha}=fc^{\alpha\beta}$ and $c^{\beta}=(1-f)c^{\alpha\beta}$, we obtain (Figs. 2, S3-S4):

$IC_{A}^{\alpha\beta}=IC_{A}^{\alpha}\frac{f}{f+(1-f)M^{\alpha\beta}}+IC_{A}^{\beta}\frac{1-f}{f/M^{\alpha\beta}+(1-f)}$,

where we have defined the motility ratio, characterizing the relative motility of bacteria in the control (Fig. S7, Dataset S7):

$M^{\alpha\beta}=\Gamma_{control}^{\beta}/\Gamma_{control}^{\alpha}$

Therefore, in the null model the combined chemotactic index $IC_{A}^{\alpha\beta}$ depends on the fraction of cells from each strain as well as their relative motility. The motility ratio was determined from the experiments used to quantify the chemotactic index of each strain in isolation (assuming the two experiments were performed for the same amount of time):

$M^{\alpha\beta}=\Gamma_{control}^{\beta}/\Gamma_{control}^{\alpha}=\frac{N_{control}^{\beta}/c^{\beta}}{N_{control}^{\alpha}/c^{\alpha}}$

**Supplementary Note 2: Null model of community composition including growth**

Equations (1) give the number of bacteria in the control and $A$-chemical-containing wells after time $T_{exp}$ (typically 1 h). We add the effect of growth for time $T_{growth}$ (usually 24 h), based on strain and substrate specific growth rates, called $\mu_{A}^{\alpha}$ (the growth rate of strain $\alpha$ on substrate $A$). Assuming exponential growth, an initial number of bacteria $N(0)$ will grow to $N(0)e^{\mu_{A}^{\alpha}T_{growth}}$. Applying this, we get:

$N_{control}^{\alpha}\left( T_{growth} \right)=e^{\mu_{A}^{\alpha}T_{growth}}\Gamma_{control}^{\alpha}T_{exp}c^{\alpha}, and N_{A}^{\alpha}\left( T_{growth} \right)=e^{\mu_{A}^{\alpha}T_{growth}}\Gamma_{A}^{\alpha}T_{exp}c^{\alpha}, N_{control}^{\beta}\left( T_{growth} \right)=e^{\mu_{A}^{\beta}T_{growth}}\Gamma_{control}^{\beta}T_{exp}c^{\beta}, and N_{A}^{\beta}\left( T_{growth} \right)=e^{\mu_{A}^{\beta}T_{growth}}\Gamma_{A}^{\beta}T_{exp}c^{\beta}.$

To obtain the community composition, we account for a fraction $f^{\alpha}$ of strain $\alpha$ and $f^{\beta}$ of strain $\beta$ (previously denoted by $f$ and $1-f$, respectively). The final fraction $F_{A}^{\alpha}$ of strain $\alpha$ on chemical $A$ is then computed as follows (Fig. 3):

$F_{A}^{\alpha}=\frac{N_{A}^{\alpha}\left( T_{growth} \right)}{N_{A}^{\alpha}\left( T_{growth} \right)+N_{A}^{\beta}\left( T_{growth} \right)}= \frac{e^{\mu_{A}^{\alpha}T_{growth}}\Gamma_{A}^{\alpha}T_{exp}c^{\alpha}}{e^{\mu_{A}^{\alpha}T_{growth}}\Gamma_{A}^{\alpha}T_{exp}c^{\alpha}+e^{\mu_{A}^{\beta}T_{growth}}\Gamma_{A}^{\beta}T_{exp}c^{\beta}} =\frac{e^{\mu_{A}^{\alpha}T_{growth}}\Gamma_{A}^{\alpha}f^{\alpha}}{e^{\mu_{A}^{\alpha}T_{growth}}\Gamma_{A}^{\alpha}f^{\alpha}+e^{\mu_{A}^{\beta}T_{growth}}\Gamma_{A}^{\beta}f^{\beta}} =\frac{f^{\alpha}}{f^{\alpha}+f^{\beta}e^{\left( \mu_{A}^{\beta}-\mu_{A}^{\alpha} \right)T_{growth}}M^{\alpha\beta}IC_{A}^{\beta}/IC_{A}^{\alpha}},$

and for strain $\beta,F_{A}^{\beta}=1-F_{A}^{\alpha}$. Note that the final composition does not change, $F_{A}^{\alpha}=f^{\alpha}$, only if the following conditions hold simultaneously: the growth rates are the same, the motility ratio is the same and the ratio of chemotactic indices is the same. If any of the three quantities change, the resulting composition will be different.

While we aimed to start with equal cell numbers in paired strain assays, achieving perfect parity was inherently challenging due to variations in cell aggregation, motility, and handling. As a result, some pairs exhibited uneven initial relative abundances (Fig. 3). This imbalance has implications for interpreting the outcomes of paired-stain assays, as a strain starting at a higher initial abundance has an inherent numerical advantage. However, the deviations we observed between experimental results and null model predictions show that inter-species interactions, rather than simple differences in starting abundances, played a dominant role in shaping final community composition. Notably, in cases where initial abundances were highly skewed, our 10% detection cut-off (Fig. S6) provides a conservative approach, potentially misclassifying real interactions as lack thereof when the initial abundance of one strain was very low. Thus, while initial abundance differences contribute to community assembly dynamics, our results highlight that inter-species effects – whether inhibitory or beneficial – can override initial numerical advantages.

**Calculation of uncertainty**

The expressions for the chemotactic index, motility index and community composition are deterministic relations. But there is a measurement of uncertainty, which can propagate into these expressions. The fundamental quantity that is measured in the ISCA is the encounter kernel $\Gamma=N/(Tc)$, where $N$ is the number of bacteria in a given well, $T$ is the time of the experiment and $c$ is the bulk concentration. Therefore, we assume that different experimental replicas are independent measurements of $\Gamma$. Assuming that the kernel $\Gamma$ follows gamma distribution (rather than normal, to avoid negative values), we estimate its mean and variance using sample mean and sample variance from the replicas. Assuming further that the measurements of different $\Gamma^{'}$ s are independent from each other, we then run Monte Carlo simulations to measure the uncertainty in $IC,M^{\alpha\beta}$ and $F_{A}^{\alpha}$ (Fig. 3), which all depend on the ratio of appropriate kernels (available on <https://doi.org/10.5281/zenodo.12210966>).

**Supplementary Figures**

**
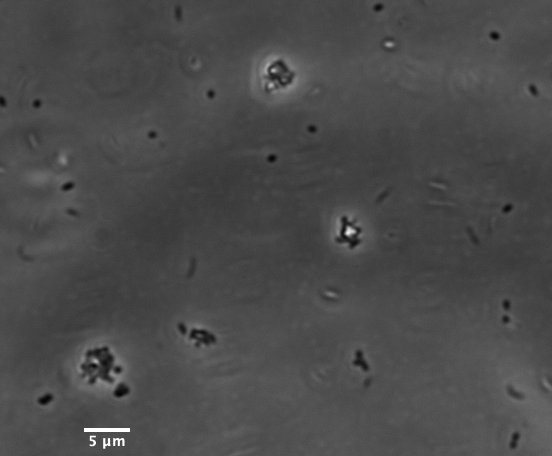
**

**Figure S1.** Light microscopy image of *Pseudoalteromonas* sp. HF66 showing the clumping phenotype. The maximum clump size was measured at 5 μm (see scale bar).


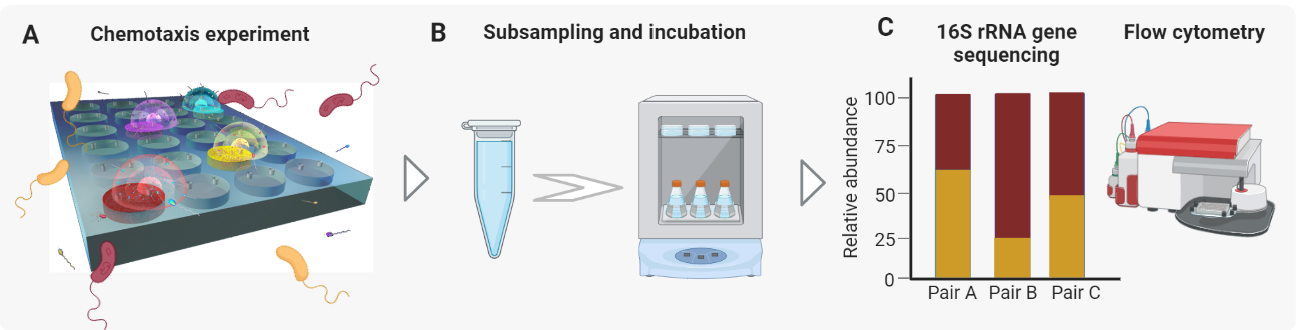


**Figure S2.** Experimental design of the chemotaxis-growth experiments. (A) Triplicate ISCAs loaded with DMSP and spermidine were incubated for 1 h in bulk artificial seawater containing a 1:1 mixture of *Pseudoalteromonas* sp. HF66 paired with one of six other bacterial strains. (B) After chemotaxis, samples were retrieved from ISCA wells and grown for 24 h. (C) Total cell abundance from the bulk artificial seawater (prior to chemotaxis), from the ISCA (after chemotaxis), and from the growth assay were determined by flow cytometry, and relative abundance of each strain was determined by DNA extraction for 16S rRNA gene amplicon sequencing.


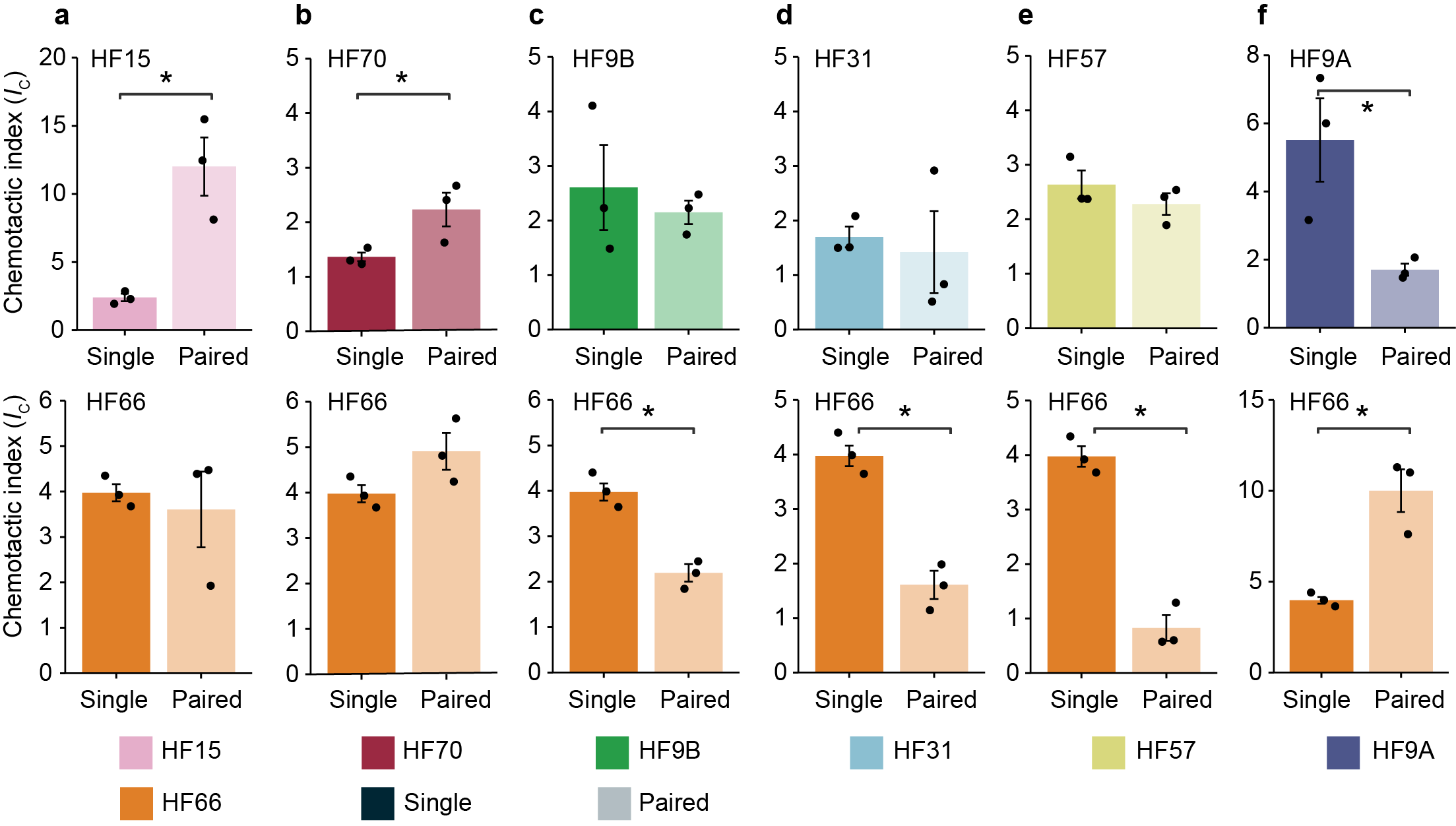


**Figure S3.** Individual chemotactic index (*I*_C_) of the strains in each pair on DMSP after 1 h of chemotaxis. Bars denote the standard deviation (*n* = 3). The experimentally determined value of *I*_C_ in chemotaxis experiments with single strains (marked as “Single”) was compared to their chemotactic performance when paired with a second strain (marked as “Paired”), namely *Pseudoalteromonas* sp. HF66 paired with *Thalassospira* sp. HF15 (a), *Vibrio* sp. HF70 (b), *Vibrio* sp. HF9B (c), *Celeribacter* sp. HF31 (d), *Vibrio* sp. HF57 (e) and *Phaeobacter* sp. HF9A (f). Individual chemotactic response of each strain in the pair was calculated from 16S rRNA gene amplicon sequencing data. Significant differences between the single and paired chemotactic indices are indicated by the asterisk (ANOVA, *P* < 0.05, Dataset S6).


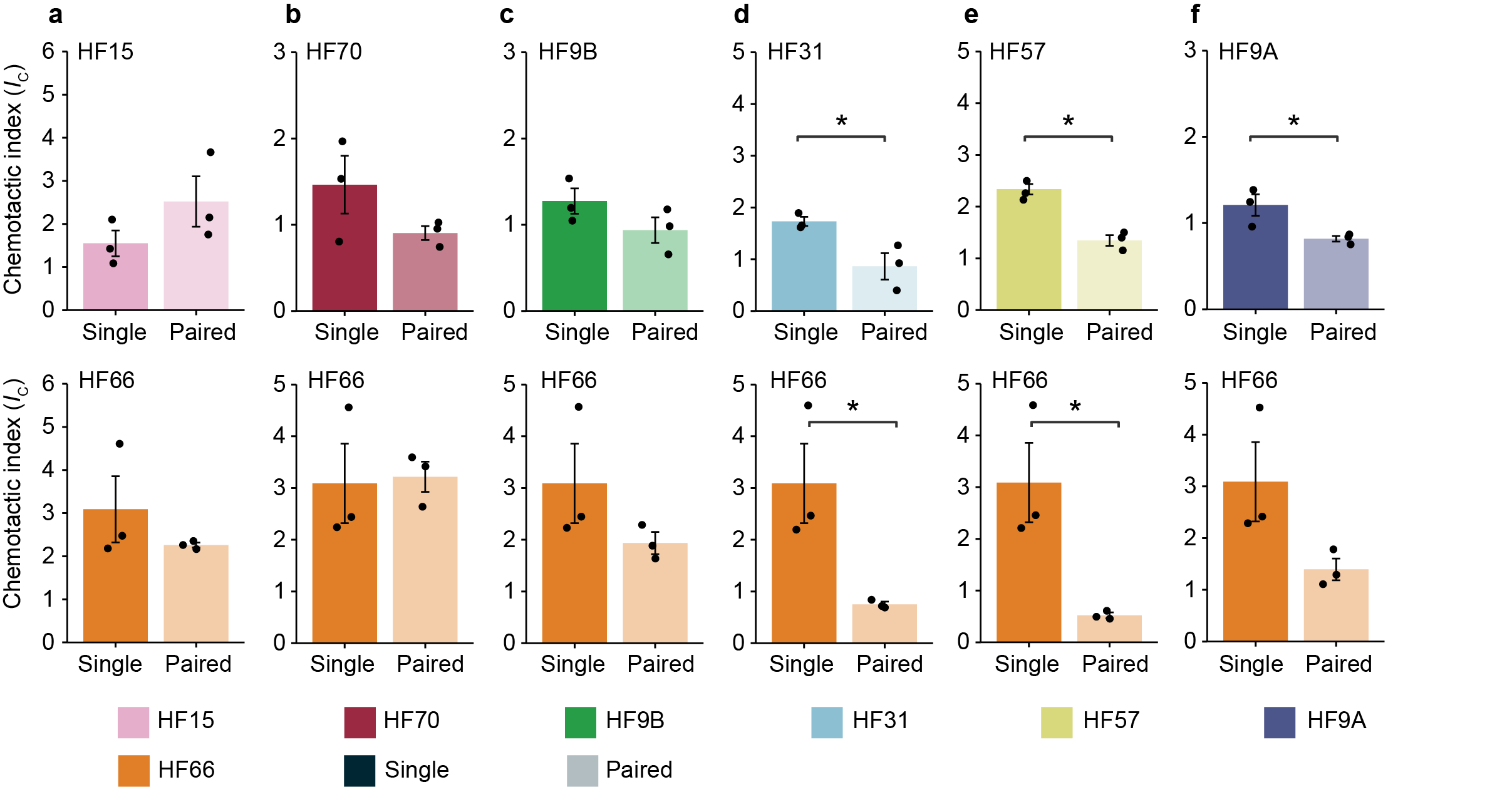


**Figure S4.** Individual chemotactic index (*I*_C_) of the strains in each pair on spermidine after 1 h of chemotaxis. Bars denote the standard deviation (*n* = 3). The experimentally determined value of *I*_C_ in chemotaxis experiments with single strains (marked as “Single”) was compared to their chemotactic performance when paired with a second strain (marked as “Paired”), namely *Pseudoalteromonas* sp. HF66 paired with *Thalassospira* sp. HF15 (a), *Vibrio* sp. HF70 (b), *Vibrio* sp. HF9B (c), *Celeribacter* sp. HF31 (d), *Vibrio* sp. HF57 (e) and *Phaeobacter* sp. HF9A (f). Individual chemotactic response of each strain in the pair was calculated from 16S rRNA gene amplicon sequencing data. Significant differences between the single and paired chemotactic indices are indicated by the asterisk (ANOVA, *P* < 0.05, Dataset S6).


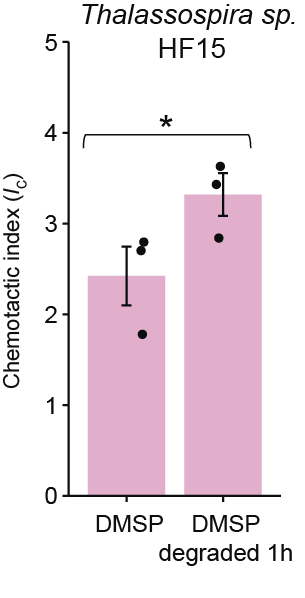


**Figure S5**. The metabolic products of *Pseudoalteromonas sp.* HF66 on DMSP affect the chemotaxis strength of *Thalassospira* sp. HF15. *Thalassospira* sp. HF15 displayed a greater chemotactic index for the spent medium of *Pseudoalteromonas* sp. HF66 incubated with DMSP than to DMSP alone. Each treatment was replicated across three different ISCAs (*n* = 3, individual dots). Data are mean ± SD. The asterisk denotes a significant difference between the two treatments (t-test, *P* ≤ 0.05, Dataset S8).


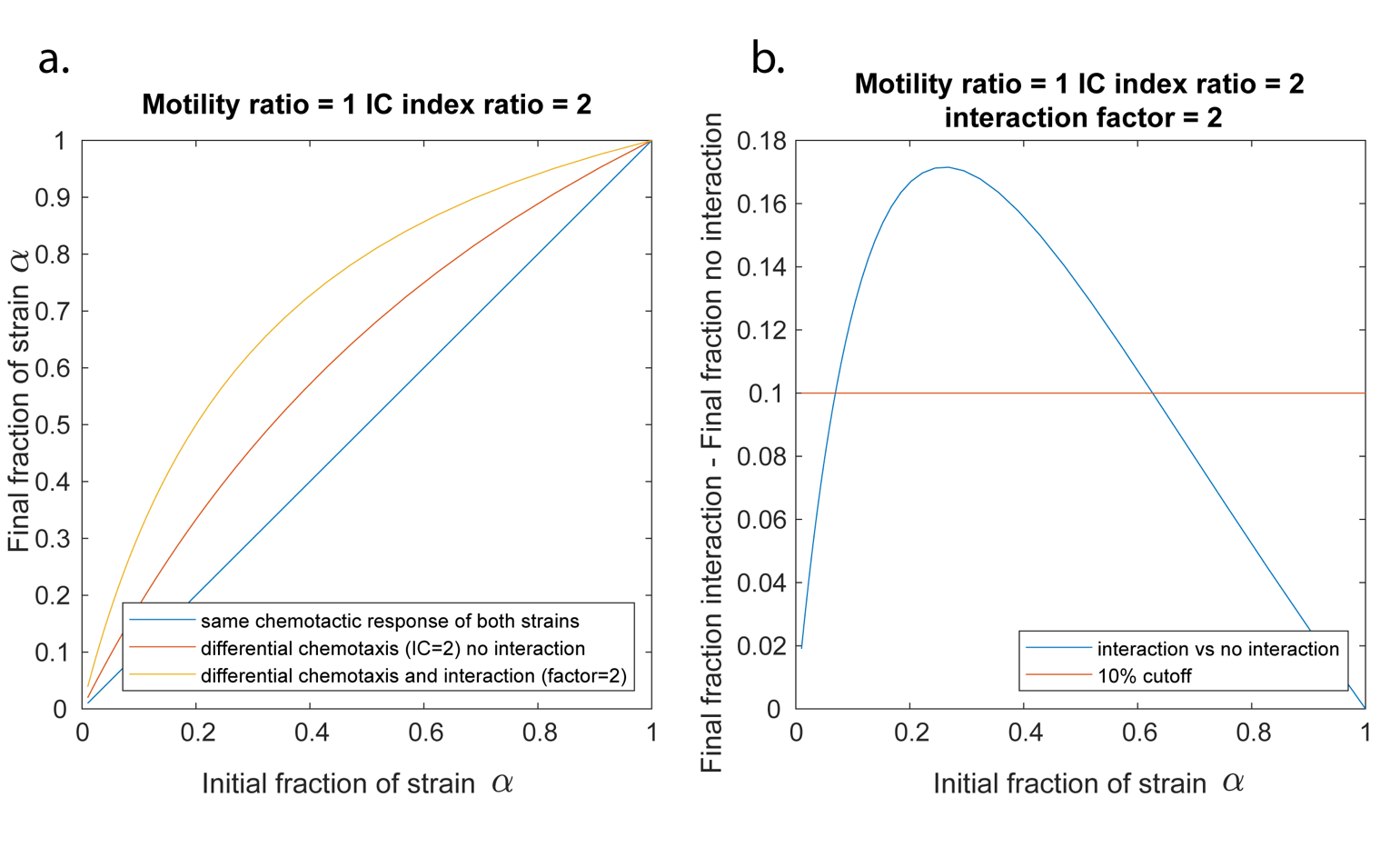


**Figure S6.** Modeling the impact of the initial fraction of one strain on its final fraction in paired chemotactic experiments. (a) Three cases are shown: (i) the two strains have identical performance (blue), (ii) one strain is a better chemotaxer than the other but the two strains do not interact (red; chemotactic index ratio = 2), and (iii) the chemotactic performance of one strain increases in the presence of the other strain (yellow; chemotactic index ratio = 4). (b) The difference in the final population fractions (yellow minus red in panel a) as a function of the initial fraction of the first strain. The red line represents the 10% cut-off we used to detect inter-species interactions.


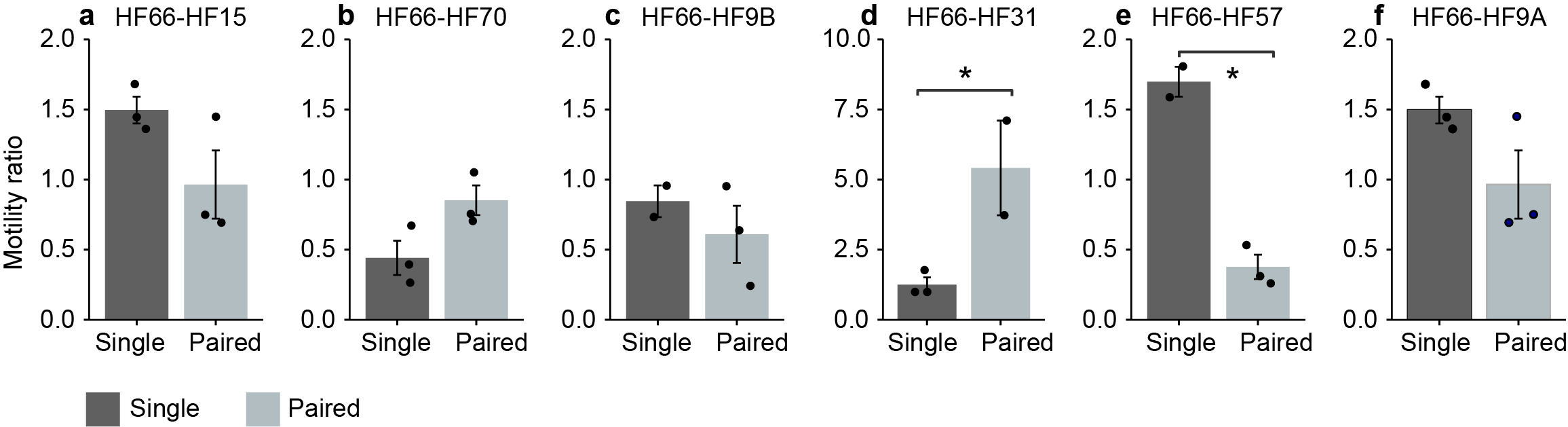


**Figure S7.** Motility ratio of *Pseudoalteromonas* HF66 and the paired strain after 1 h of chemotaxis. Bars denote the standard deviation (*n* = 3). The model-predicted motility ratio with single strains (marked as “Single”) was compared to their observed motility ratio when paired with a second strain (marked as “Paired”), namely *Pseudoalteromonas* sp. HF66 paired with *Thalassospira* sp*.* HF15 (a), *Vibrio hepatarius* sp. HF70 (b), *Vibrio diazotrophicus* sp. HF9B (c), *Celeribacter* sp*.* HF31 (d), *Vibrio* sp. HF57 (e) and *Phaeobacter* sp. HF9A (f). Motility ratio in each pair was calculated from 16S rRNA gene amplicon sequencing data. Significant differences between the single and paired ratios are indicated by the asterisk (ANOVA, *P* ≤ 0.05, Dataset S7).

**Datasets (separate file)**

**Dataset S1.** Welch two sample t-tests with significance levels for the growth responses on all test metabolites. Significance of growth on each metabolite was calculated by comparing OD_600_ = 24h to OD_600_ = 1h. Data are displayed in Fig. 1 and 4a-g.

**Dataset S2.** Welch two sample t-tests with significance levels for the chemotactic responses on DMSP. Significance (*P* ≤ 0.05) was calculated by comparing strength of the response to DMSP with the negative control of filtered artificial seawater (SW). Data are displayed in Fig. 1a.

**Dataset S3.** Welch two sample t-tests with significance levels for the chemotactic responses on spermidine. Significance (*P* ≤ 0.05) was calculated by comparing strength of the response to spermidine with the negative control of filtered artificial seawater (SW). Data are displayed in Fig. 1b.

**Dataset S4**. Sums of squares (SS), mean squares (MS) and significance levels for the analyses of variance (ANOVAs) of the observed and predicted chemotactic responses of paired strains, reported in Fig. 2 (diff: differences in mean; lower and upper: confidence intervals).

**Dataset S5**. Cell counts derived from two-strain communities competition experiment determined by 16S rRNA amplicon sequences. Cell counts were calculated from raw reads using mock microbial communities of known cell densities. Data are displayed in Fig. 3.

**Dataset S6**. Sums of squares (SS), mean squares (MS) and significance levels for the analyses of variance (ANOVAs) of the single and paired chemotactic responses of the strains, reported in Fig. S3-S4 (diff: differences in mean; lower and upper: confidence intervals).

**Dataset S7**. Welch two sample t-tests with significance levels for the motility ratio of paired and single strains (*P* ≤ 0.05). Data are displayed in Fig. S7.

**Dataset S8**. Welch two sample t-tests with significance levels for the chemotactic responses of HF15. Significance (*P* ≤ 0.05) was calculated by comparing strength of the response to DMSP with supernatant containing DMSP-degradation products generated by HF66. Data are displayed in Fig. S5.

**Dataset S9**. Percent deviation between the predicted (model) and observed (amplicon) community composition. Deviations greater than 10% are highlighted in red.

**Dataset S10**. Welch two sample t-tests with significance levels for the chemotactic responses on all metabolites. Significance (*P* ≤ 0.05) was calculated by comparing the strength of the response to each metabolite with the negative control of filtered artificial seawater (SW). Data are displayed in Fig. 4.
